# A nineteen-year report of serotype and antimicrobial susceptibility of enteric non-typhoidal *Salmonella* from humans in Southern India: changing facades of taxonomy and resistance trend

**DOI:** 10.1186/s13099-020-00388-z

**Published:** 2020-10-23

**Authors:** Jobin John Jacob, Dhanalakshmi Solaimalai, Dhiviya Prabaa Muthuirulandi Sethuvel, Tanya Rachel, Praveena Jeslin, Shalini Anandan, Balaji Veeraraghavan

**Affiliations:** Department of Clinical Microbiology, Christian Medical College and Hospital, Vellore, Tamil Nadu 632004 India

**Keywords:** Non-typhoidal *Salmonella*, Multidrug resistance, Surveillance, Serovar

## Abstract

**Background:**

The steady increase in the proportion of Non-typhoidal *Salmonella* (NTS) infections in humans represents a major health problem worldwide. The current study investigated the serovar distribution and antimicrobial susceptibility trends of NTS isolated from faecal samples during the period 2000–2018.

**Methods:**

Faecal specimens of patients were cultured according to standard lab protocol. The isolates were serotyped and antimicrobial susceptibility testing (AST) were performed according to CLSI guidelines.

**Results:**

A total of 1436 NTS isolates were obtained from faeces samples mostly comprising of *S.* Typhimurium (27.3%), *S.* Weltevreden (13%), *S.* Bareilly (11%), *S.* Newport (4.2%), *S*. Cholerasuis (4%), *S.* Infantis (3.4%), and *S*. Enteritidis (2.4%). Resistance to nalidixic acid (26%) was most common among the tested NTS, followed by ampicillin (18.5%), cotrimoxazole (13.5%), ciprofloxacin (12%), ceftriaxone (6.3%) and chloramphenicol (3.6%). Multidrug resistance was observed in 5% of NTS isolates with the highest rate (10.52%) in 2014. The incidence of NTS infection was maximum in children < 5 years of age with an average 19.3% of the total affected patients during the time period.

**Conclusions:**

Based on this study, the faecal NTS isolates have high resistance rates against first line antimicrobial agents except chloramphenicol. The gradual but consistent increase in resistance to fluoroquinolones, third generation cephalosporins and macrolide may restrict future treatment options. Hence periodic monitoring of NTS infections, serotype distribution and antimicrobial resistance trend is recommended.

## Introduction

Non typhoidal *Salmonella* (NTS) is frequently associated with diarrheal illness or self-limiting gastroenteritis in humans around the world [1]. Global estimates suggest 93.8 million cases of infection and 155,000 deaths annually [2]. A greater proportion of human NTS infections are often related to the consumption of contaminated food or water [3]. NTS can also be spread via the faecal—oral route or by direct contact with infected animals [4]. Unlike typhoidal *Salmonella* (*Salmonella* Typhi and *Salmonella* Paratyphi) NTS infections primarily reported in developed countries [5]. In developing countries the incidence of NTS causing gastroenteritis remains poorly documented and the burden in public health has not been identified [6].

Based on the antigenic variations (O, H_1_, H_2_ and Vi) *Salmonella enterica* is classified into over 2,500 serotypes in which almost 1531 serovars belong to host specific, clinically relevant *Salmonella enterica* subsp. enterica [7]. The serotypes have great influence on the severity of the NTS infections as well as the host in which they cause disease [1]. For instance *S*. Dublin is associated with enteric disease in cattle, *S*. Gallinarum in chicken, *S*. Choleraesuis in swine, *S*. Typhimurium and *S*. Enteritidis in a wide range of hosts [8]. However recent reports suggest many of these serovars are capable of crossing the intestinal epithelial layer and cause invasive disease in humans [9].

The use of antimicrobial therapy has not been recommended for most of the NTS infections. However, treatment is recommended in the severely ill (bacteremia and meningitis) or immunocompromised patients (HIV) [10]. The emergence of antimicrobial resistant isolates restricted the therapeutic options and increased the disease burden in a global scale [11]. The global establishment of antibiotic resistant NTS isolates is associated with the uncontrolled use of antibiotics in livestock animals [12]. Treatment with third generation cephalosporins or fluoroquinolones was found to be effective previously. Nonetheless resistance to cephalosporins and fluoroquinolones along with first line antimicrobial agents is increasing at an alarming rate [13].

Despite the increasing prevalence of NTS infections, the epidemiology of NTS has not been completely understood in India. Limited studies have been conducted to establish the epidemiology of NTS in humans. Knowledge on the serotype distribution and antibiotic susceptibility patterns is important to formulate appropriate therapeutic and control strategies. In this study, we investigated the serotype diversity, age distribution and antimicrobial resistant trend of NTS isolated from human faecal samples during the period 2000 to 2018 in a tertiary care hospital in Southern India. The data presented here represent all NTS isolates from faecal sample isolated between 2000 and 2018. The samples include both hospitalized (inpatient) and community-based samples (outpatient).

## Materials and methods

### Isolation and identification of NTS

Faecal specimens of patients having clinical suspicion of gastroenteritis were received by the clinical microbiology lab in Christian Medical College, Vellore, India, for a period of 19 years (2000–2018). The samples were processed according to World Health Organization (WHO) Global Foodborne Infections Network laboratory protocol [14]. Colonies suspected as *Salmonella* sp. were Gram stained, sub-cultured and identified by standard biochemical tests. Serogroup of NTS isolate was confirmed by slide agglutination with commercial typing antiserum (Becton-Dickinson, CA, USA) followed by the Kauffmann-White scheme [15]. NTS isolates were sent to the National *Escherichia* and *Salmonella* Center (NESC), Kasauli, India for antigenic profiling and serovar identification. A standard case report form containing queries about demographic, clinical, and epidemiologic information of the patient was recorded as part of the study.

### Antimicrobial susceptibility testing

Antimicrobial susceptibility test (AST) for ampicillin (10 µg), chloramphenicol (30 µg), trimethoprim/sulfamethoxazole (25 µg), nalidixic acid (30 µg), ciprofloxacin (5 µg), ceftriaxone (30 µg) and azithromycin (15 µg) was performed using disk diffusion method. Experiments were performed and the results were interpreted as per the Clinical and Laboratory Standards Institute CLSI, 2016 guidelines for azithromycin and CLSI, 2018 for other antibiotics [16, 17]. *Escherichia coli* ATCC 25,922, *Enterococcus faecium* ATCC 29,212, and *Pseudomonas aeruginosa* ATCC 27,853 was used as quality control strain for antimicrobial susceptibility testing. Strains were considered to be multidrug resistant (MDR) if they were resistant to all three first line antibiotics (ampicillin, chloramphenicol, and cotrimoxazole) or at least to three classes of antimicrobials.

### Data analysis

All obtained data was entered into Microsoft Excel spreadsheet. Descriptive data is given as bar graphs, line graphs and tables. Chi-squared analysis was performed to determine the trends in the proportion of NTS isolates from period 1 and period 2. A *p*-value < 0.05 was considered statistically significant.

## Results

**Fig. 1:**
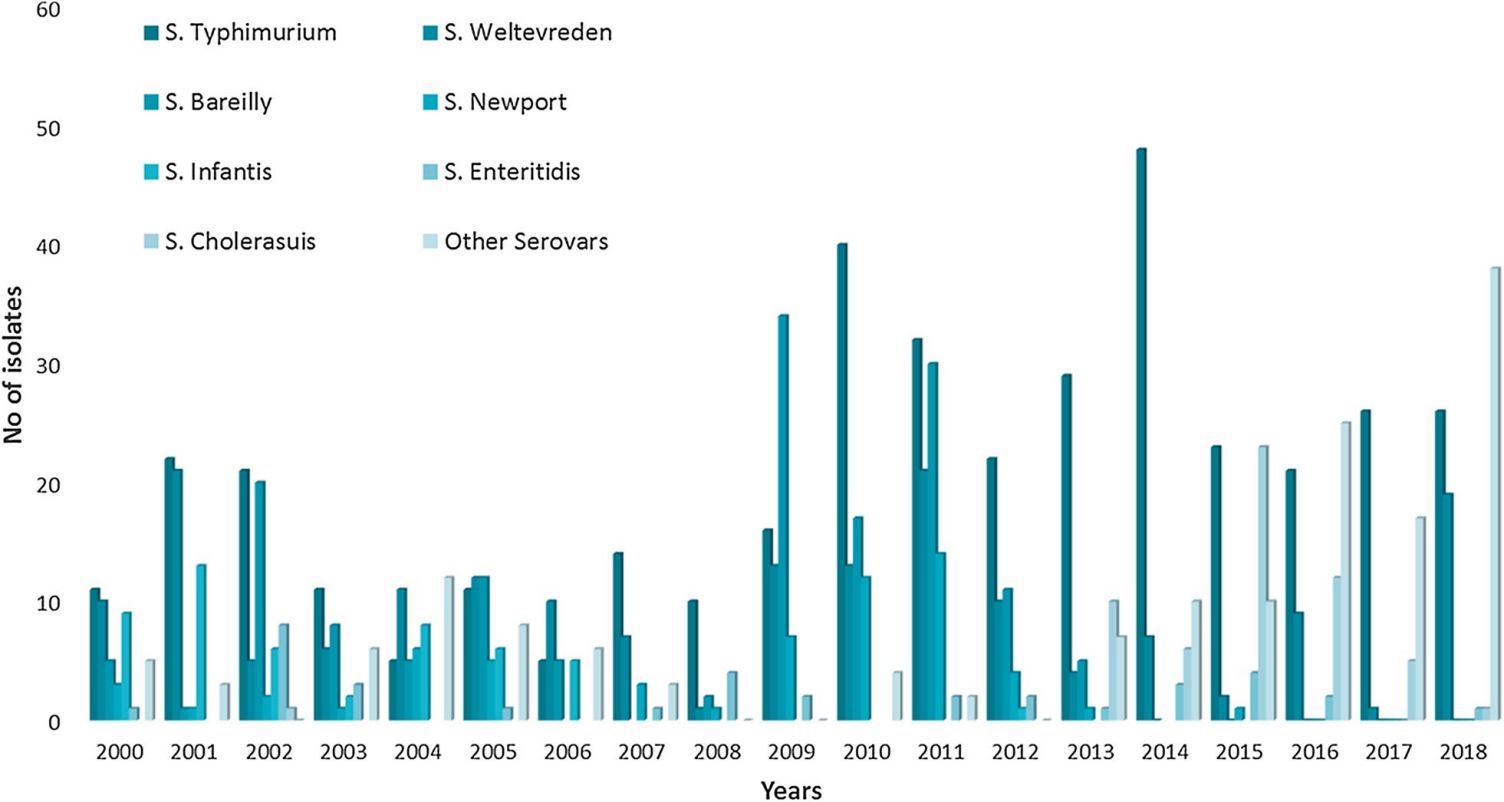
Year wise distribution of invasive non-typhoidal *Salmonella* serovars identified from India during the period 2000–2018

A total of 1436 NTS isolates were identified during January 2000 to December 2018 from faecal specimens collected at tertiary care hospital after the exclusion of duplicates. Year wise comparison of the number of NTS isolates suggests a sudden increase from the year 2009 (Fig. 1). An average of 29% NTS isolates was identified from the first 8 years (2000–2008) whereas the incidence increased to 71% in the next 10 years. Among the 1436 isolates, *S*. Typhimurium found to be the most prevalent serotype with 393 isolates (27.3%). The second and third most prevalent serovars of NTS in the study area were *S.* Weltevreden and *S.* Bareilly which accounted for 13% (182/1436) and 11% (155/1436) respectively (Fig. 1). While the isolation of *S.* Typhimurium was found to be consistent through the years, the prevalence of *S.* Weltevreden and *S.* Bareilly was inconsistent although declining over the years. The prevalence of other major serovars are as follows *S.* Newport (61, 4.2%), *S.* Infantis (50, 3.4%), *S*. Enteritidis (35, 2.4%) and *S.* Cholerasius (58, 4%). *S.* Lindenberg were only detected from the year 2015, 2015 (4/93), 2016 (25/95), 2017 (12/90) and 2018 (1/153) but not seen before. The other 27 detected serovars accounted for 7% (101/1,436) of the total number of isolates. Unusual serovars were also identified such as *S.* Hassarek, *S.* Eastbourne, *S.* Ughelli, *S.* Abortusequi and so on.

**Fig. 2:**
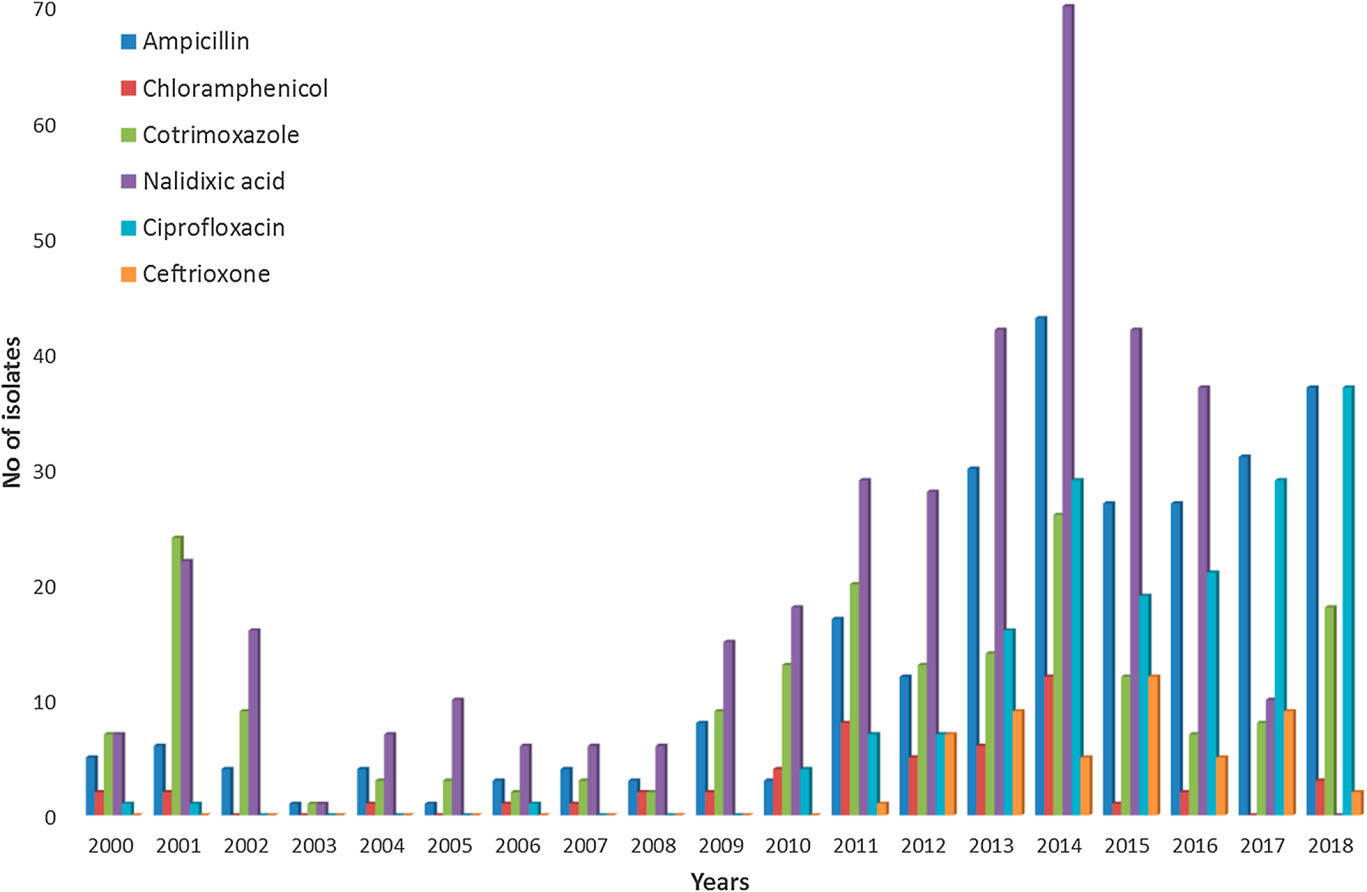
Year wise distribution of antimicrobial resistance against clinically relevant antimicrobials by NTS isolates identified from 2000–2018

The antibiotic resistance has been increasing in most of the clinically important bacteria. Resistance to nalidixic acid (27%) was most common among the tested NTS, followed by resistance to ampicillin (19.3%), cotrimoxazole (14%), ciprofloxacin (12.4%) and chloramphenicol (4%). Ceftriaxone susceptibility testing done from 2011 to 2018 showed that 6.2% (50/794) of the isolates were resistant and there was in increasing trend in resistance through the years. Similarly, azithromycin susceptibility done in 2017 and 2018 showed resistant rate of 6% and 8% respectively. Year wise antimicrobial resistance pattern of NTS isolates are shown in Fig. 2. From the data we have observed there was a significant increase in antimicrobial resistance profile to fluoroquinolones (ciprofloxacin) and third generation cephalosporins (ceftriaxone) from 2010 to 2018. Hence based on changes in antimicrobial resistance the data has been represented in two study periods (Table 1).

**Table 1:** Antimicrobial resistance pattern of NTS isolates between 2000–2009 and 2010–2018

Antibiotics	2000–2018 n = 1436	2000–2009 n = 495	2010–2018 n = 941	*P* value
Ampicillin	18.5 (266/1436)	7.9 (39/495)	24.1 (227/941)	0.005
Chloramphenicol	3.6 (52/1436)	2.2 (11/495)	4.3 (41/941)	0.439
Cotrimoxazole	13.5 (194/1436)	12.7 (63/495)	13.9 (131/941)	0.813
Ciprofloxacin	12 (172/1436)	0.60 (3/495)	17.9 (169/941)	< 0.001
Ceftrioxone	6.3 (50/794)	Not tested	6.3 (50/794)	> 0.99
Nalidixic acid	26 (372/1436)	19.4 (96/495)	29.3 (276/941)	0.124

**Fig. 3:**
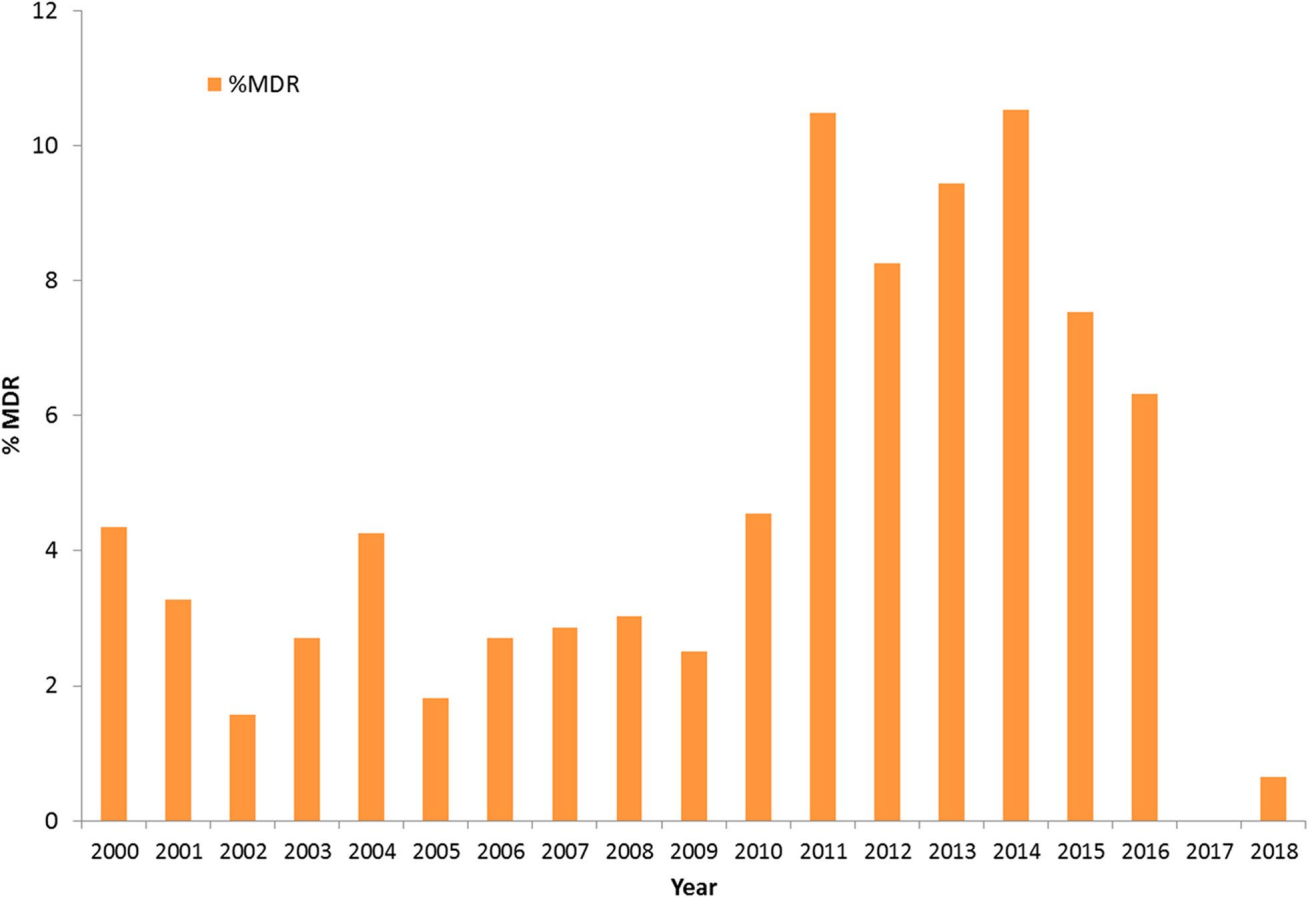
Year wise distribution of Multidrug resistant NTS isolates identified from 2000–2018

Multidrug resistance (resistant to all three first line antibiotics (ampicillin, chloramphenicol, and cotrimoxazole) or at least to three classes of antimicrobials was observed in 5% (73/1436) of NTS isolates with the highest rate (10.52%) in year 2014 (Fig. 3). *S.* Typhimurium was the predominant MDR serotype (33/73) followed by *S.* Bareilly (9/73), *S.* Weltevreden (7/73) and *S.* Newport (4/73). The untypable isolates accounted for 14.1% (10/73) of the total MDR isolates and *S.* Cholerasius (n = 3) *S.* Infantis (n = 2), *S.* Senftenberg (n = 1), *S.* Chester (n = 1) and *S.* Lindenburg (n = 1) were the other detected MDR serovars.

**Fig. 4:**
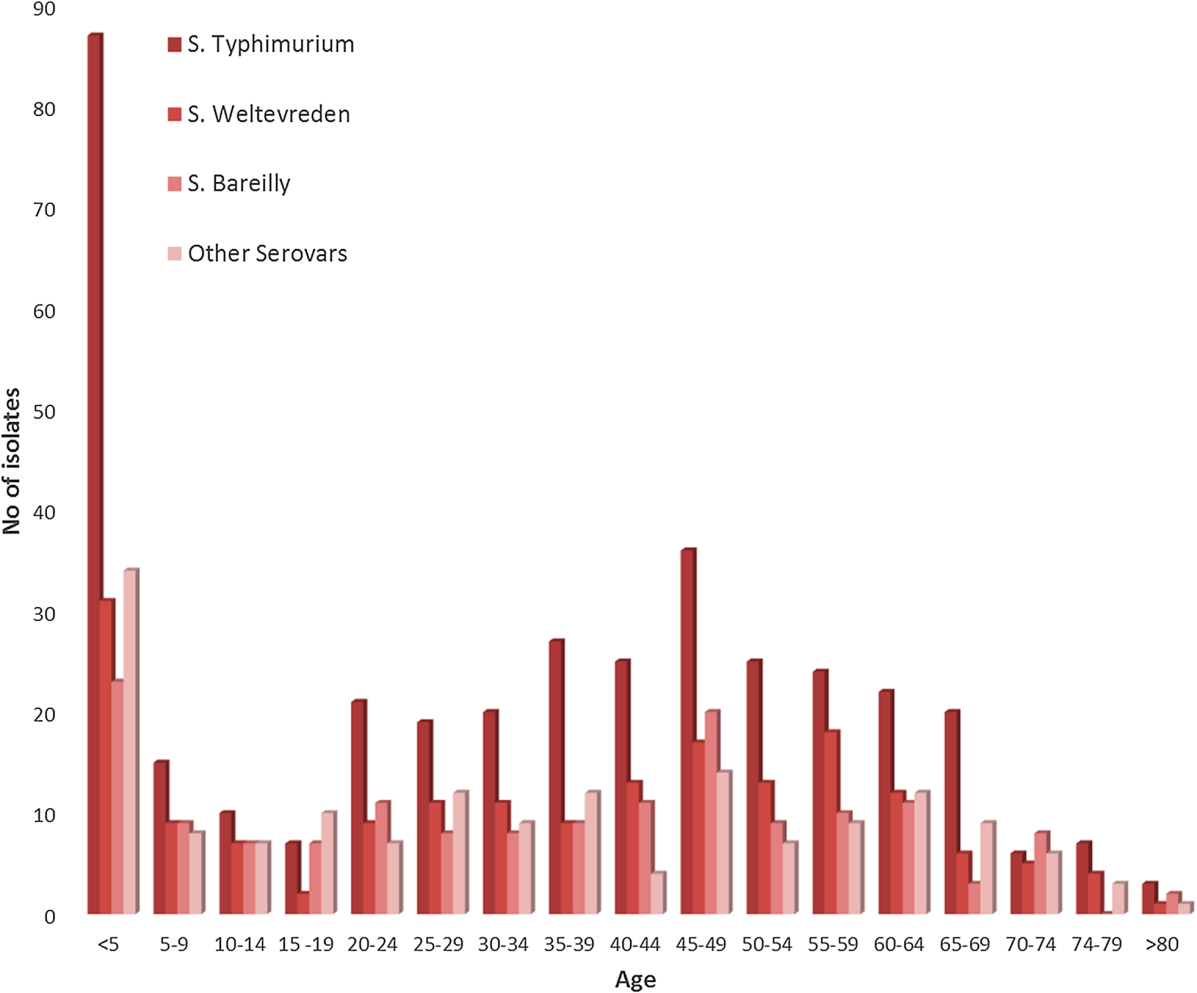
The age based distribution of patients having NTS infection from 2000–2018

The age distribution of patients having NTS infection through the years (2000–2018) is shown in Fig. 4. The incidence of NTS infection was maximum in children < 5 years of age which is an average of 19.3% the total affected patients during the time period. The overall age specific isolation rates of NTS were 4.7% (5–9 yrs), 3.5% (10–14 yrs), 2.9% (15–19 yrs), 5.5% (20–24 yrs), 5.7% (25–29 yrs), 5.5% (30–34 yrs), 6.5% (35–39 yrs), 6% (40–44 yrs), 9.9% (45–50 yrs), 6.1% (50–54 yrs), 6.9% (55–59 yrs), 6.5% (60–64 yrs), 4.3% (65–69yrs), 2.8% (70–74 yrs) and 0.8% (> 80 yrs). The male-to-female ratio of NTS infection was approximately 1.5:1.

## Discussion

*Salmonella enterica* represents the most pathogenic species of global public health significance and is the leading cause of foodborne illnesses in humans and animals worldwide [18]. However, NTS infections is often neglected or rarely reported in India [19]. On the other hand, there is a scarcity of data on the occurrence of NTS infections including serovar distribution and antimicrobial susceptibility profile in the country. In this study we have examined the serovar distribution and antimicrobial resistance pattern of 1436 NTS strains isolated from faecal samples during the period 2000–2018.

The year wise distribution of NTS serovars from human faeces samples showed an increasing trend after the year 2008. The sharp increase in the number of NTS isolates suggests the improved surveillance or a greater number of faecal samples processed in the study centre. Previous study from India showed that *S.* Weltevreden, *S.* Typhimurium and *S.* Bareilly were reported to be the three most common human NTS serotype isolated [20]. Whilst, *Salmonella* Typhimurium was the most frequently identified serovar followed by *S.* Weltevreden, *S.* Bareilly, *S.* Newport, *S.* Infantis, *S.* Enteritidis and *S.* Cholerasius in this study. Although *S.* Enteritidis is said to be the most commonly isolated serovar from humans globally [21], this study indicated that *S.* Enteritidis ranked seventh in the frequency of isolation. In contrast previous studies done in NTS bloodstream infections (iNTS) were in agreement with the global report as *S.* Enteritidis were the second common invasive serotype after *S.* Typhimurium (Unpublished data; Table 2). The variation in NTS serovar distribution may be due to the changes in geographical or environmental factors in developed and developing countries [21]. In general the proportion of *S.* Typhimurium to *S.* Enteritidis in south India follows the trend as in developed countries.

**Table 2:** Non-typhoidal *Salmonella enterica* serovars associated with invasive disease, 2000 to 2018, India

*Salmonella* serovar	iNTS n (%)	NTS n (%)	Total (N)	Relative risk of invasiveness compared to *S. Typhimurium* (95% CI)	*p* value
*S. Typhimurium*	140 (26.27)	393 (73.73)	533	NA	
*S. Enteritidis*	84 (71.19)	34 (28.81)	118	2.22 (1.80,2.73)	< 0.001
*S. Bareilly*	17 (9.9)	155 (90.1)	172	0.16 (0.08,0.32)	< 0.001
*S. Weltevreden*	9 (5.26)	182 (94.74)	191	0.16 (0.08,0.32)	< 0.001
*S. Cholerasius*	5 (7.94)	58 (92.06)	63	0.36 (0.15,0.83)	0.017
*S. Dublin*	4 (40)	6 (60)	10	1.27 (0.58,2.75)	0.55
*S*. Cerro	5 (33.33)	10 (66.67)	15	0.84 (0.36,1.98)	0.698
*S. Infantis*	4 (7.4)	50 (92.6)	54	0.221 (0.07,0.67)	0.007
*S. Newport*	2 (3.17)	61 (96.83)	63	0.10 (0.03,0.41)	0.001
*S. Kentucky*	11 (26.83)	30 (73.17)	41	3.17 (2.70,3.72)	< 0.001
*S. Gallinarium*	2 (100)	0	2	3.17 (2.70,3.72)	< 0.001
*S. Hadar*	1 (100)	0	1	3.17 (2.70,3.72)	< 0.001
*S. Derby*	1 (10)	9 (90)	10	0.24 (0.04,1.61)	0.143
*S. Lindenberg*	2 (6.45)	29 (93.54)	31	1.06 (0.34,3.31)	0.926

The important features that makes NTS different from typhoidal includes (a) NTS have broad host range, mostly host adapted (human and animals) and causes infection worldwide in contrast to typhoidal *Salmonella* which are host (human) restricted and are endemic to developing countries (b) NTS have stable genome than typhoidal *Salmonella* which tends to degrade (c) NTS has the strong ability to acquire AMR genes from other enteric bacteria in the gut where the possibility of the lateral gene transfer is high [22, 23]. These highlights the significance of NTS in the human infections.

The changing trends in serotype distribution of NTS through the years may influence the clinical manifestations. In this study the distribution of *S.* Typhimurium was consistent or stable, however the number of *S.* Weltevreden and *S.* Bareilly have significantly decreased over the years. However this pattern was not established among the NTS isolates from blood specimen (Table 2). *S.* Enteritidis (Serogroup D) that has been reported to be invasive has a good distribution in bloodstream infections while *S.* Cholerasius (Serogroup C1) is found to be prevalent in human faecal samples.

Antimicrobial resistance in NTS isolates was relatively lower when compared to studies from other countries [24]. The isolates exhibited higher antimicrobial resistance to first line antibiotics (ampicillin and co-trimoxazole) except chloramphenicol. Resistance to ampicillin showed a marginal increase until 2014. Resistance to co-trimoxazole and nalidixic acid also showed similar trends between the years 2003 and 2014 with highest level of resistance observed in 2014 and 2001 to co-trimoxazole and nalidixic acid respectively. As a result of the increasing resistance, the first line antibiotics are no longer used for the treatment of NTS infections [25]. Fluoroquinolone or extended spectrum cephalosporins are currently used in the therapeutics for NTS infections. However, the gradual but steady increase in resistance to these therapeutic agents raises concerns regarding the control of pathogenic NTS serovars [26]. Based on changes in overall antibiotic resistance the study period was divided into two in which the resistance rates found to be significantly increased during period 2.

The prevalence of MDR isolates have steady risen worldwide. Our study showed that multidrug resistance has increased from 1.5% in 2002 to 10.5% in 2014. The number of MDR isolates was higher in period 2 (2009–2018) compared to period 1 (2000–2008). Hence the increase in MDR isolates in recent years is in agreement with the global reports [27]. Overall reports from other parts of India showed high levels of antimicrobial resistance to nalidixic acid, ampicillin/amoxicillin, cotrimoxazole and ciprofloxacin in NTS isolates when compared to that in our study [28–30].

The antimicrobial resistance of NTS isolates based on serotype distribution at different age groups showed that this data is consistent with studies from other parts of the world [31]. Global reports suggest that the highest risk for NTS was in infants and this study follows the similar trend. About 19.3% of NTS infections were in patients aged < 5 and the prevalence of NTS infections was found to be decreasing with age. The overall NTS was also high in adults between the ages 45–49. This study also showed that the male to female ratio of NTS disease (1.5:1) also correlated with the reports from other parts of the world [32].

The number of infections caused by faecally transmitted pathogens is still challenging in developing countries [33]. The prevalence of human cases of gastroenteritis can be many times higher than the number of reported and confirmed cases. Although relatively low levels of antimicrobial resistance were observed in our study, it is also evident that the overall antimicrobial resistance and multidrug resistance are on the rise over the years. The increasing trend in antimicrobial resistance can be associated with the wide use of antibiotics in animals and plant products [34]. Based on this study, the faecal NTS isolates have high resistance rates against first line antimicrobial agents except chloramphenicol. The gradual but consistent increase in resistance to fluoroquinolones and third generation cephalosporins suggests that NTS infections should be closely monitored. In addition, resistance to azithromycin, one of the clinically important antimicrobial seems to be increasing but not significantly in this study.

Furthermore, reports on carbapenem and colistin resistance in NTS cannot be ignored although these drugs are not first-choice for the treatment of *Salmonella* infections. Carbapenems and colistin are considered as the last resort antibiotics for the treatment of infections caused by MDR Gram-negative pathogens in humans [35, 36]. The emergence of resistance to these antibiotics in zoonotic pathogens such as NTS poses a pressing threat to public health, as they can transferred to humans through the food chain. Although carbapenem resistance is still very rare in NTS, various carbapenemase-encoding genes were identified in non-typhoidal serovars of *Salmonella* enterica isolated in humans from different countries including India. This includes, Cubana, Typhimurium, Waycross, Senftenberg, Westhampton, Stanley, Agona, Kentucky, and Saintpaul [23].

With regards to colistin, though there is no clinical relevance, resistance has been reported in *S. enterica* from food producing animals. This could be due to the wide use of colistin in animal production for therapeutic, prophylactic and growth promotion purposes in several countries [37]. Plasmid mediated colistin resistance (*mcr*) is the most commonly reported mechanism which spreads by lateral transfer to commensals or pathogens of animal and human origin. These resistance genes in NTS could be derived from other nosocomial enteric bacteria particularly *K. pneumoniae* and *E. coli* through lateral gene transfer as these genes are commonly located on mobile genetic elements which can efficiently contribute to their spread [23]. However, carbapenem and colistin susceptibility was not done for the study isolates and thus not discussed.

The clinical presentation of NTS infections such as HIV and malaria in adults and malnutrition in children have not been taken into account. Other limitations of this study include the lack of data on antibiotics exposure within patients before sampling. The limitation of disc diffusion in the antibiotic sensitivity testing is another shortcoming of this study. The inherent drawbacks of serovar prediction based on slide agglutination method (White-Kauffmann-Le Minor scheme) found to be a major limitation in this study. Depending slide agglutination as the only method for serovar identification may not be reliable as phase conversion and microevolution in antigen genes could give incorrect results for closely related serovars [40]. This is in line with the molecular characterisation of serogroup B serovars from the same study centre as traditional serotyping yielded major discrepancies with an accuracy of only 85%. A number of *S*. Typhimurium isolates from this study were predicted as *S.* Agona or *S.* Saintpaul (Serogroup B) after MLST based molecular typing [41]. This shows that molecular typing techniques are needed for the accurate prediction of *Salmonella* serovars based on the sequence data. Alternatively, Whole Genome Sequencing (WGS) based method or CRISPR based typing methods are being reported for accurate identification of NTS serovars [38] But concerns regarding the cost associated with sequencing and the technical proficiency required should be duly considered.

In conclusion, the serotype distribution and overall antimicrobial resistance trend of human NTS isolates from India showed a similar trend to that of sub-Saharan African countries or Southeast Asian countries. The presence of significant number of potentially invasive *Salmonella* serotypes of serogroup C1 and serogroup D suggest a prospective rise in clinical infections by NTS in India. The slow emergence of antibiotic resistance rates and multidrug resistant phenotypes warrants the continuous monitoring of susceptibility trend of NTS in India. However increasing resistance to fluoroquinolones and third generation cephalosporins may restrict future treatment options. Also, it is of extreme importance to monitor the susceptibility of carbapenem and colistin in NTS in order to save these last resort drugs. Epidemiological surveillance of AMR is required to establish possible links between reservoirs and to limit the lateral transfer of the resistance genes between *S. enterica* and other bacteria. Hence periodic monitoring of NTS infections, serotype distribution and antimicrobial resistance is recommended.

## Data Availability

Not applicable.
